# Prospective mortality in patients with non-fatal deliberate self-harm: a national cohort study

**DOI:** 10.1192/j.eurpsy.2024.169

**Published:** 2024-08-27

**Authors:** P. Qin

**Affiliations:** National Center for Suicide Research and Prevention, University of Oslo, Oslo, Norway

## Abstract

**Introduction:**

Deliberate self-harm (DSH) is a strong indicator of psychological distress and constitutes a significant risk factor for subsequent mortalities.

**Objectives:**

In this study we want to gain insights into cause-specific mortalities in self-harming patients and to disentangle important factors differentiating the risks so that to inform follow-up care and mortality prevention.

**Methods:**

Retrospective data from nationwide registries were interlinked to follow all patients presenting to specialist healthcare with non-fatal DSH from January 2008 through December 2018. Data on cause of death, personal socioeconomic status, clinical features of DSH and other medical covariates were retrieved. The Fine and Gray competing risks model was used to identify significant factors impacting subsequent mortality risk by specific causes of death in the cohort.

**Results:**

The cohort of 43153 DSH patients comprised 24286 females and 18867 males, with 45.3% being 10-34 years old, 38.1% being 35-64 years old and 16.6% above 65 years old at index DSH episode. Of these patients, 7041 died during the follow-up period, including 2290 within the first 1-year, corresponding to a mortality rate of 31.9 per 1000 person-years in the follow-up period and 54.9 per 1000 person-years in the first year. Common causes of death included suicide (n=911), other external causes (n=1020), cancer (n=896), cardiovascular diseases (n=1523), respiratory disease (n=787) and mental and substance misuse disorders (n=463), but the causes of death varied greatly by age groups and other factors. The risk of dying by suicide was highly associated with middle-age, male gender, tertiary education, psychiatric history, and DSH by injury, clear intent of self-harm, comorbid affective or personality disorder, referral to psychiatric treatmen, as well as DSH repetition during the period of follow-up. Significant risk factors for death by other external causes included male gender, old or middle age, single marital status, lowest quartile income, history of psychiatric treatment, and DSH by injury and comorbid substance misuse. For death by natural causes, the relative risk was highest among the elderly and the middle-aged, with other significant risk factors including male gender, single marital status, low education, lowest quartile income, and comorbid substance misuse. Attendance in psychiatric treatment after DSH appeared to be beneficial reducing the risk for mortality by suicide, other external causes and natural causes as well.

**Conclusions:**

Patients with DSH represent a high-risk group for suicide, other external and natural cause mortalities. Mental healthcare is essential in follow-up care and personalized care should take into account patients´ socio-demographic background and clinical features of self-harm.

**Disclosure of Interest:**

None Declared

